# Author Correction: Metagenome-assembled genomes and gene catalog from the chicken gut microbiome aid in deciphering antibiotic resistomes

**DOI:** 10.1038/s42003-022-04266-z

**Published:** 2022-11-24

**Authors:** Yuqing Feng, Yanan Wang, Baoli Zhu, George Fu Gao, Yuming Guo, Yongfei Hu

**Affiliations:** 1 State Key Laboratory of Animal Nutrition, College of Animal Science and Technology, China Agricultural University, 100193 Beijing, China; 2 College of Veterinary Medicine, Henan Agricultural University, 450046 Zhengzhou, Henan China; 3 CAS Key Laboratory of Pathogenic Microbiology and Immunology, Institute of Microbiology, Chinese Academy of Sciences, 100101 Beijing, China

**Keywords:** Microbiome, Metagenomics, Metagenomics

Correction to: *Communications Biology* 10.1038/s42003-021-02827-2, published online 18 November 2021.

In the original version of this article, several references were incorrectly numbered in the text. These have now been corrected as follows:

On Page 2, 10 changes have been made to correctly cite references 7, 11, 12, 13, 14, 15, 16, 17 and 18–21.

On Page 3, 2 changes have made to correctly cite references 7, 18 and 19.

On Page 4, 1 change has been made to correctly cite reference 7.

On Page 6, 3 changes have been made to correctly cite reference 7, 11 and 13.

In addition, 4 references were listed out of order in the references list. These have now been corrected to:

8. Broom, L. J. & Kogut, M. H. The role of the gut microbiome in shaping the immune system of chickens. *Vet. Immunol. Immunopathol*. **204**, 44–51 (2018).

9. Gordon, J. I., Raoult, D. & Henrissat, B. The abundance and variety of carbohydrate-active enzymes in the human gut microbiota. *Nat. Rev. Microbiol*. **11**, 497–504 (2013).

10. Segura-Wang, M., Grabner, N., Koestelbauer, A., Klose, V. & Ghanbari, M. Genome-resolved metagenomics of the chicken gut microbiome. *Front. Microbiol*. **12**, 726923 (2021).

11. Stewart, R. D. et al. Compendium of 4941 rumen metagenome-assembled genomes for rumen microbiome biology and enzyme discovery. *Nat. Biotechnol*. **37**, 953–961 (2019).

This has now been corrected in the PDF and HTML versions of the Article.

