# Supplementary material for: Metagenome-assembled genomes and gene catalog from the chicken gut microbiome aid in deciphering antibiotic resistomes
Source: Commun Biol. 2021 Nov 18;4:1305. doi: 10.1038/s42003-021-02827-2 (PMC8602611; doi:10.1038/s42003-021-02827-2)
Supplement: Supplementary file 2 — Supplementary Information [file 42003_2021_2827_MOESM2_ESM.pdf]

# Supplementary Figure 1

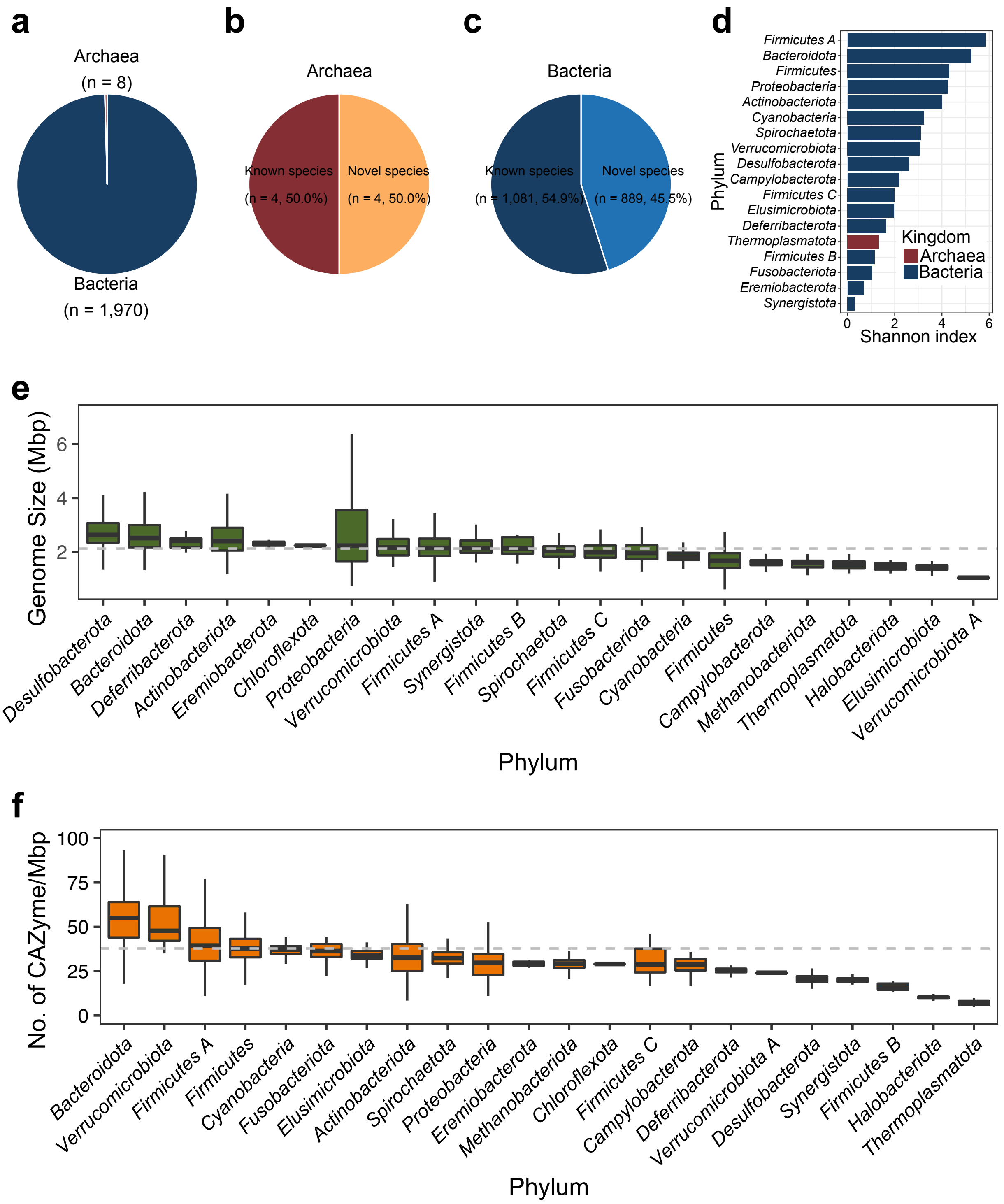


**Supplementary Figure 1: Statistics of the MAGs. (a) Number of bacteria and archaea at the species level. (b) Number of novel archaeal species. (c) Number of novel bacterial species. (d) Diversity of strains in each phylum. The diversity was calculated using the following formula: H = . (x: the number of strains in one species). (e) Genome size of MAGs in different phyla. (f) Number of CAZymes per million bp of each genome in different phyla.**

# Supplementary Figure 2

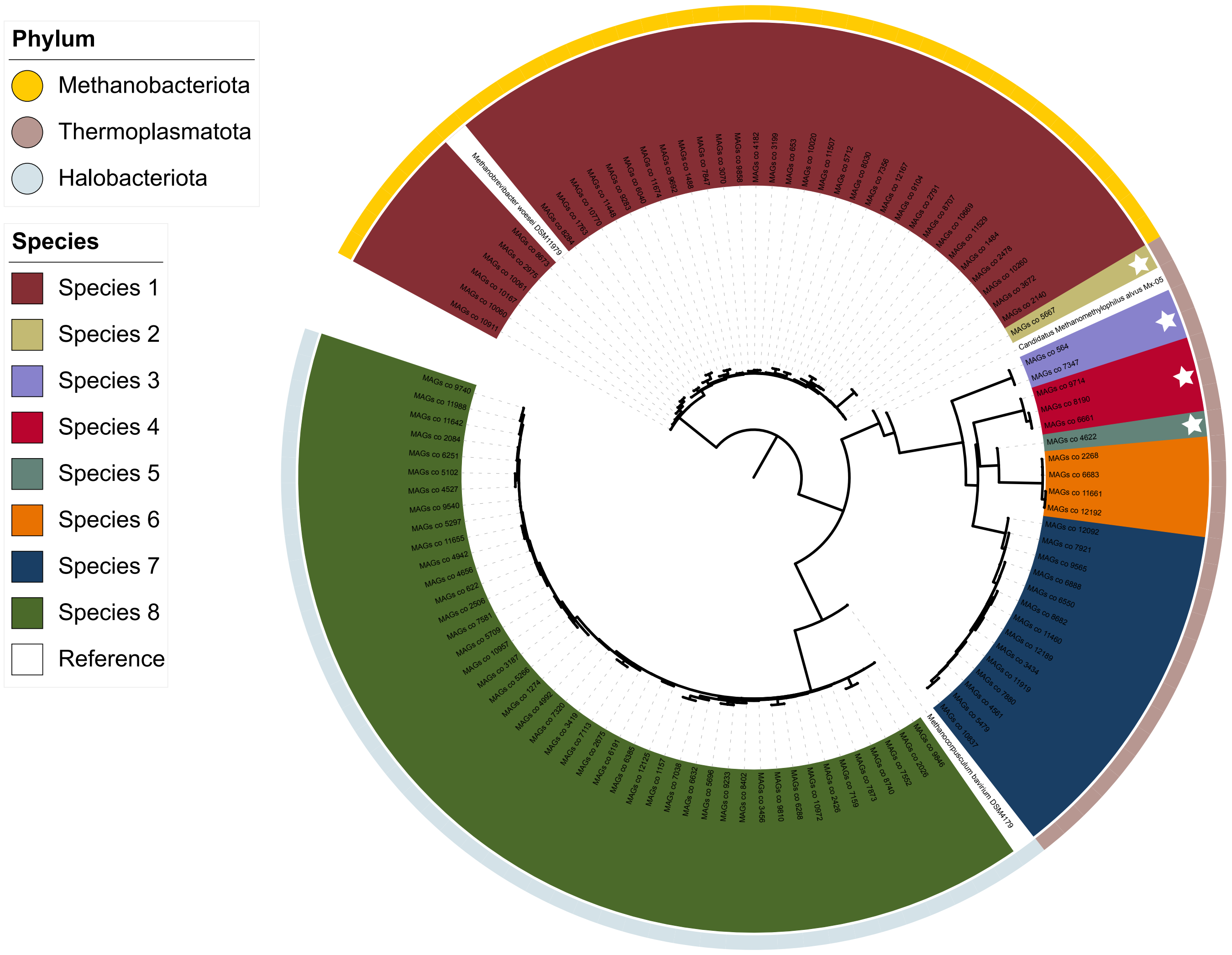


**Supplementary Figure 2: Phylogenetic tree of 107 archaeal MAGs (99% ANI) and three reference genomes. The different colored ranges represent the different species. The outer ring represents the phylum. The white stars represent novel species.**

# Supplementary Figure 3

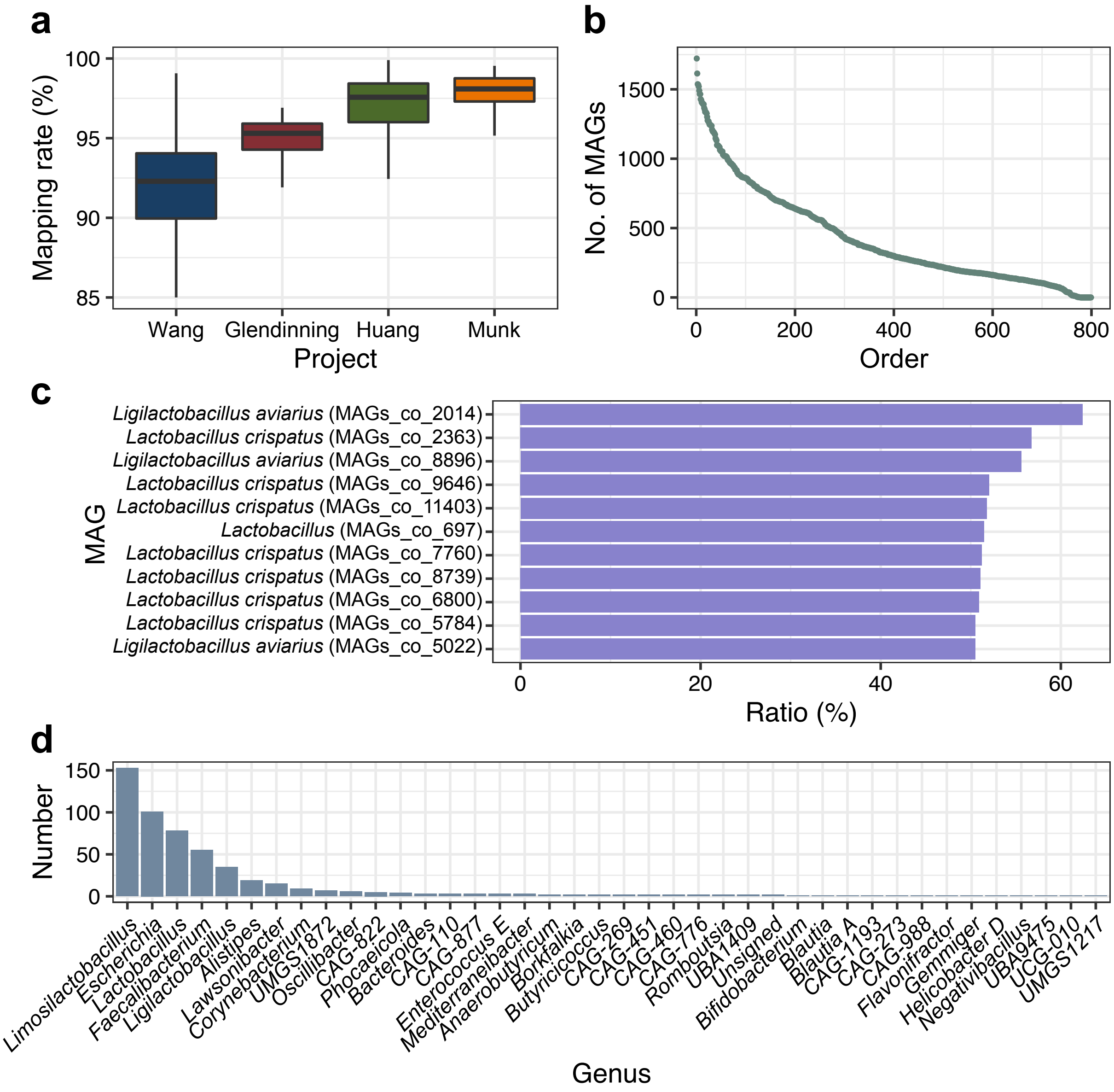


**Supplementary Figure 3: Distribution of the 12,339 MAGs. (a) Metagenomic read mapping rates in the four sequencing projects (see Materials and Methods) against the 12,339 MAGs. (b) Number of MAGs presented in the 799 samples with over 1 × coverage. (c) List of MAGs found in more than half of all 477 samples with over 15 million paired-end sequencing reads. (d) The number of MAGs in each genus among the 535 strain-level MAGs shared by samples from all ten countries.**

# Supplementary Figure 4

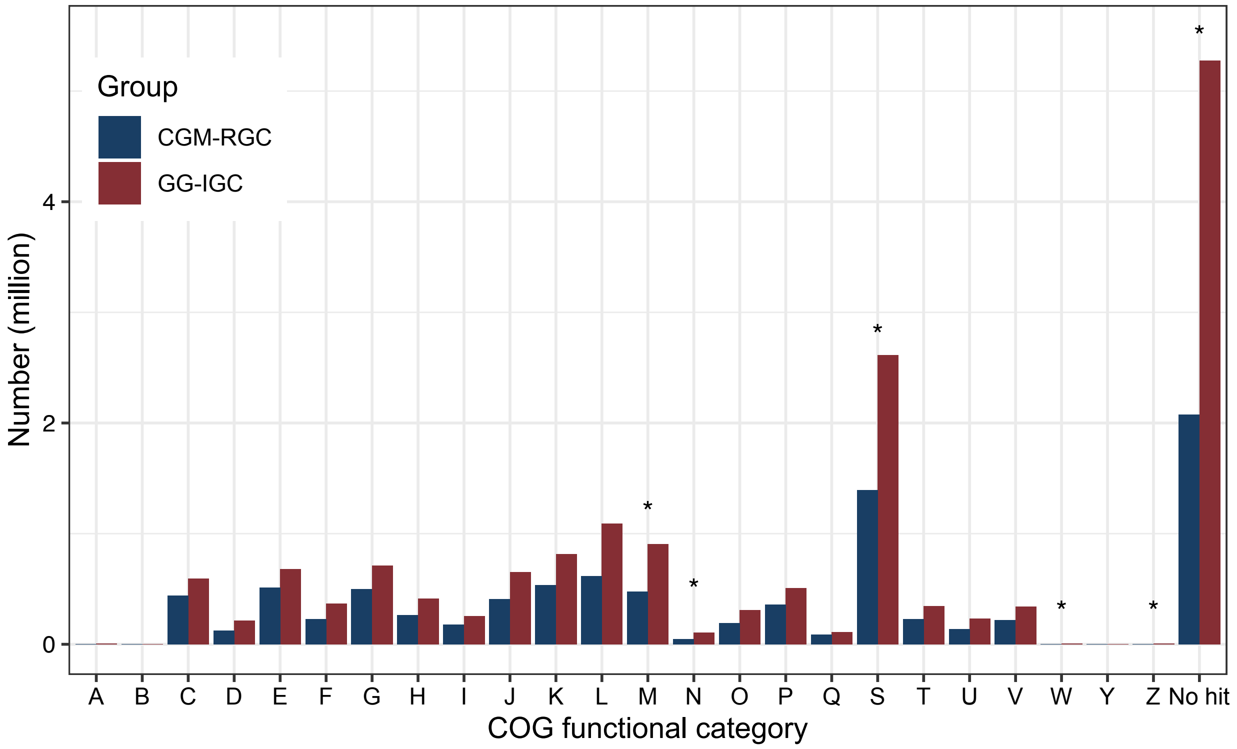


**Supplementary Figure 4: Numbers of genes assigned to different COG functional categories. *, Ratio of the GG-IGC to the CGM-RGC in each COG functional category over the ratio of the number of genes from the two gene catalogs (1.83, 16,565,684/9,037,241).**

# Supplementary Figure 5

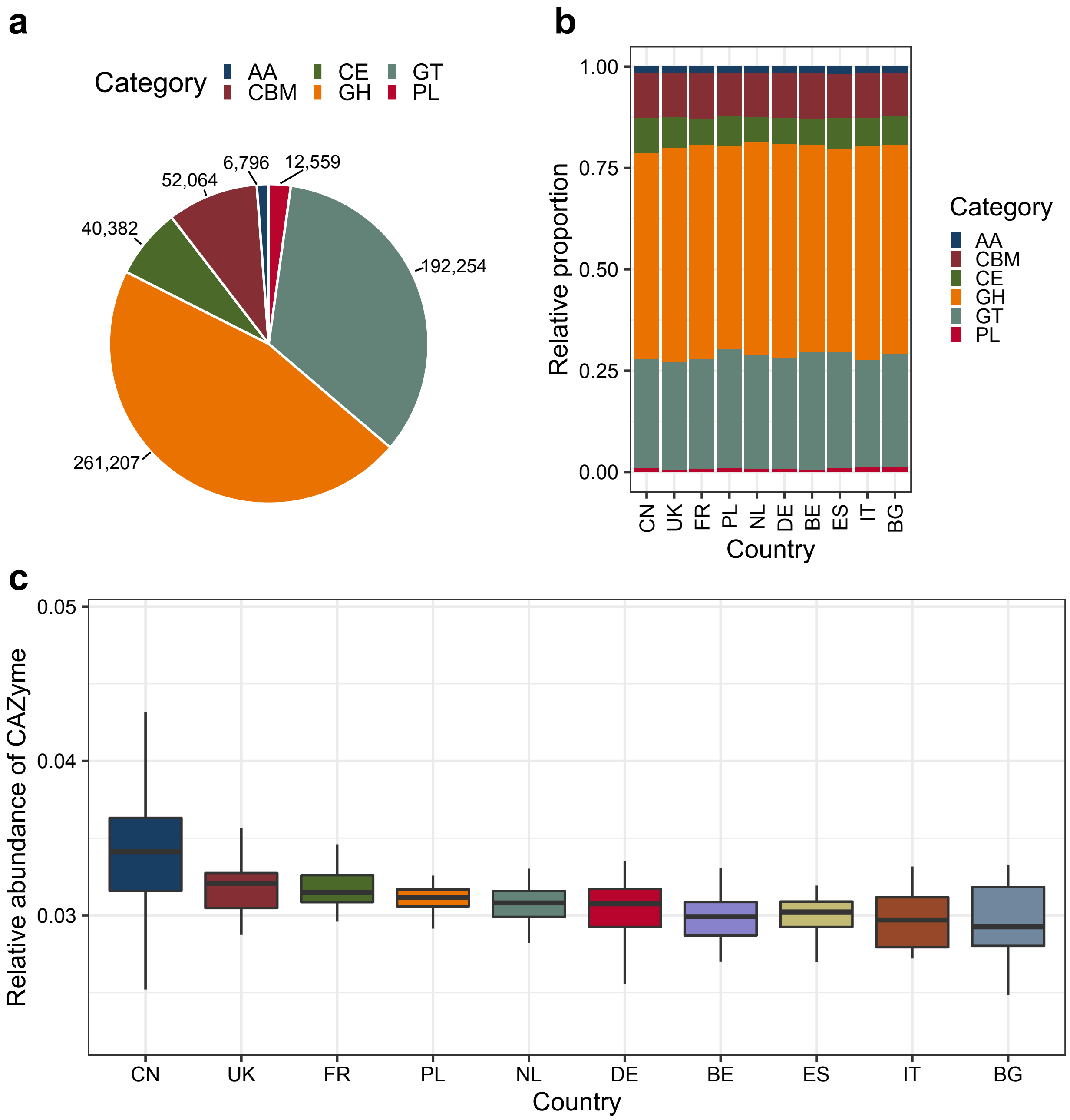


**Supplementary Figure 5: Abundances and categories of CAZymes in 477 samples with over 15 million paired-end reads from different countries. (a) Relative abundance of CAZymes in the samples from the ten countries. (b) Number of CAZymes in each category. AA: Auxiliary activity; CBM: Carbohydrate-binding module; CE: Carbohydrate esterase; GH: Glycoside hydrolase; GT: Glycosyltransferase; PL: Polysaccharide Lyase. (c) Relative proportions of different categories of CAZymes in samples from different countries.**

# Supplementary Figure 6

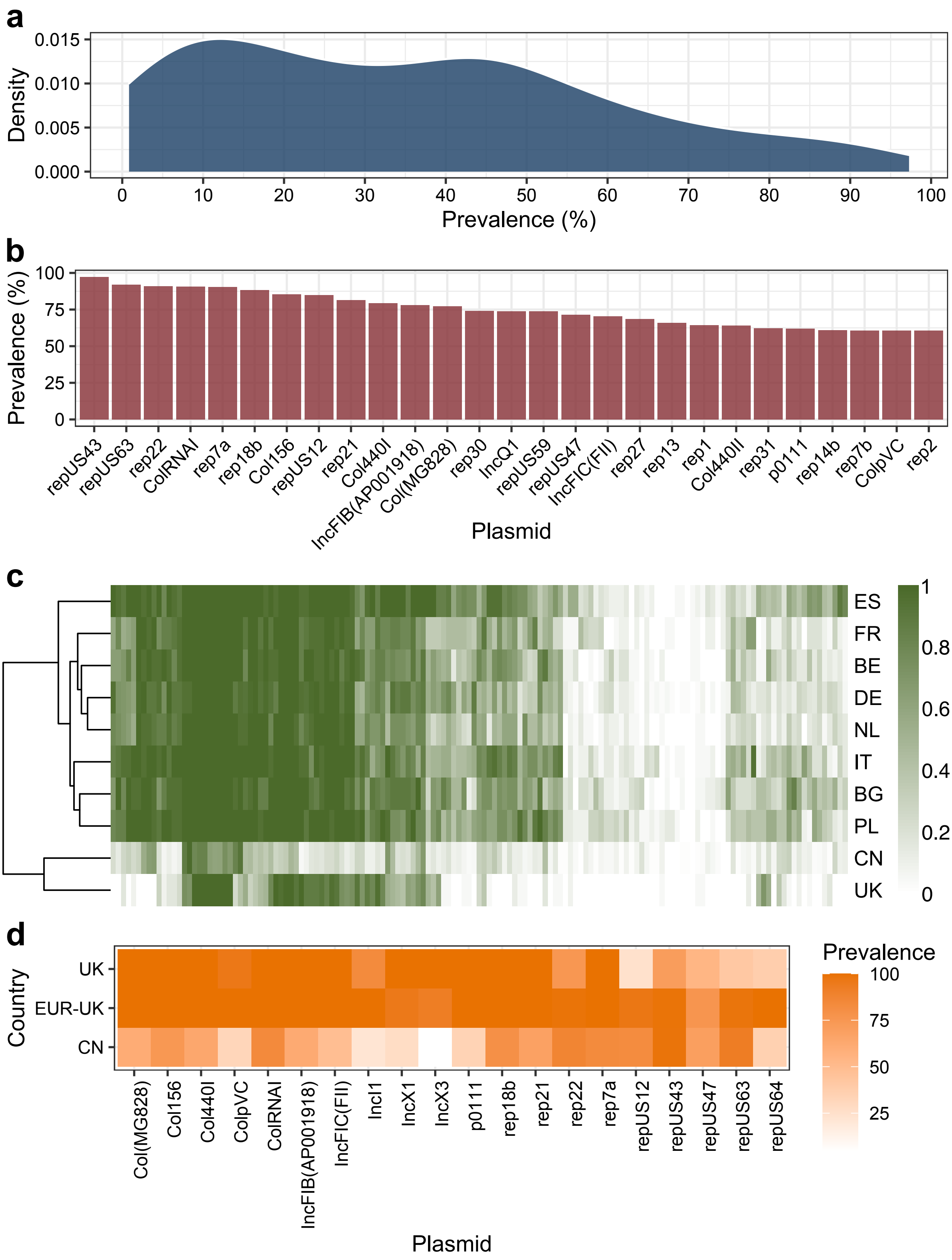


**Supplementary Figure 6: Distribution of plasmids in the 477 samples. (a) Prevalence of plasmids found in the gene catalog. (b) Plasmids distributed in over 60% of the 477 samples. (c) Heatmap of the distribution of plasmids in samples from the ten countries. (d) Top 10 most prevalent plasmids in samples from China, the UK and other countries in Europe except the UK.**

# Supplementary Figure 7

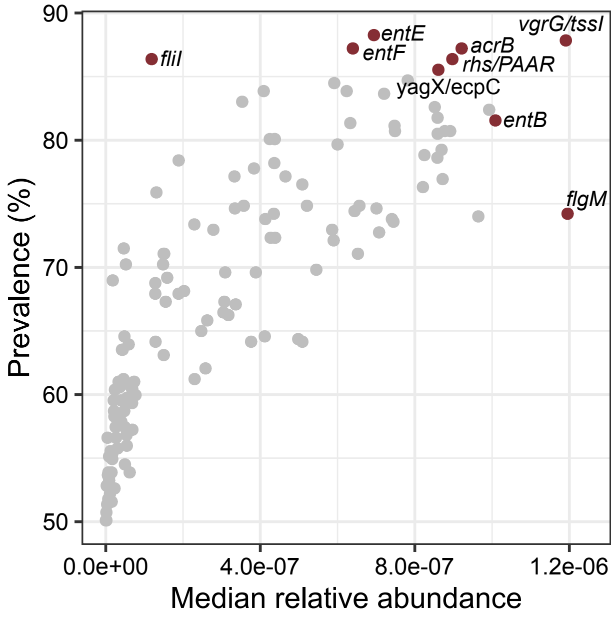


**Supplementary Figure 7: Median relative abundances of virulence factor genes distributed in over 50% of the 477 samples.**

# Supplementary Figure 8

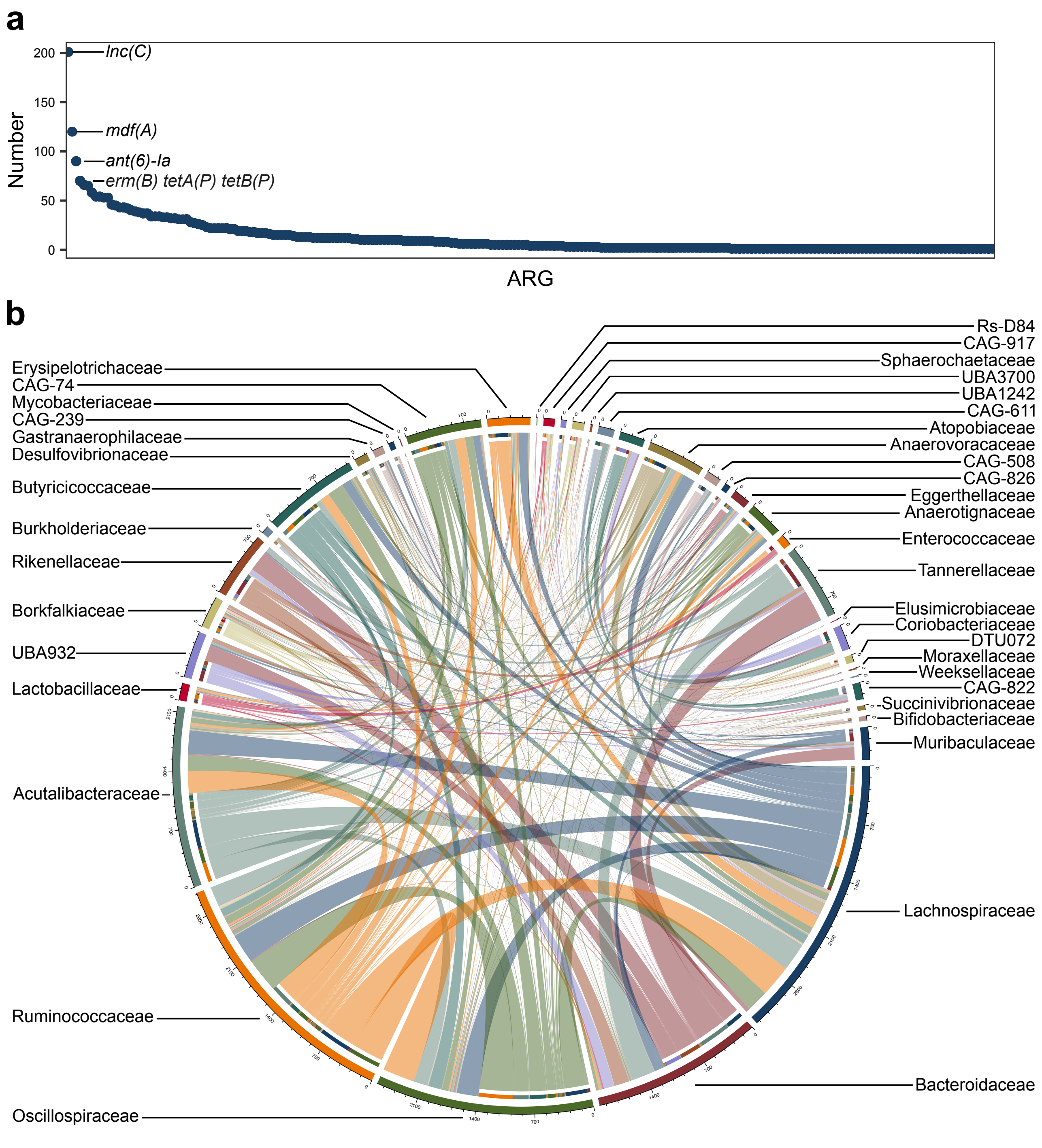


**Supplementary Figure 8: ARGs in MAGs and HGT analysis. (a) Dot plot of the total number of ARGs found in the 12,339 MAGs. (b) Predicted gene flow within the chicken gut microbiome at the family level. The bands connect donors and recipients, with the width of the band corresponding to the number of HGTs and the color corresponding to the donor. The inner bands indicate the recipients.**

# Supplementary Figure 9

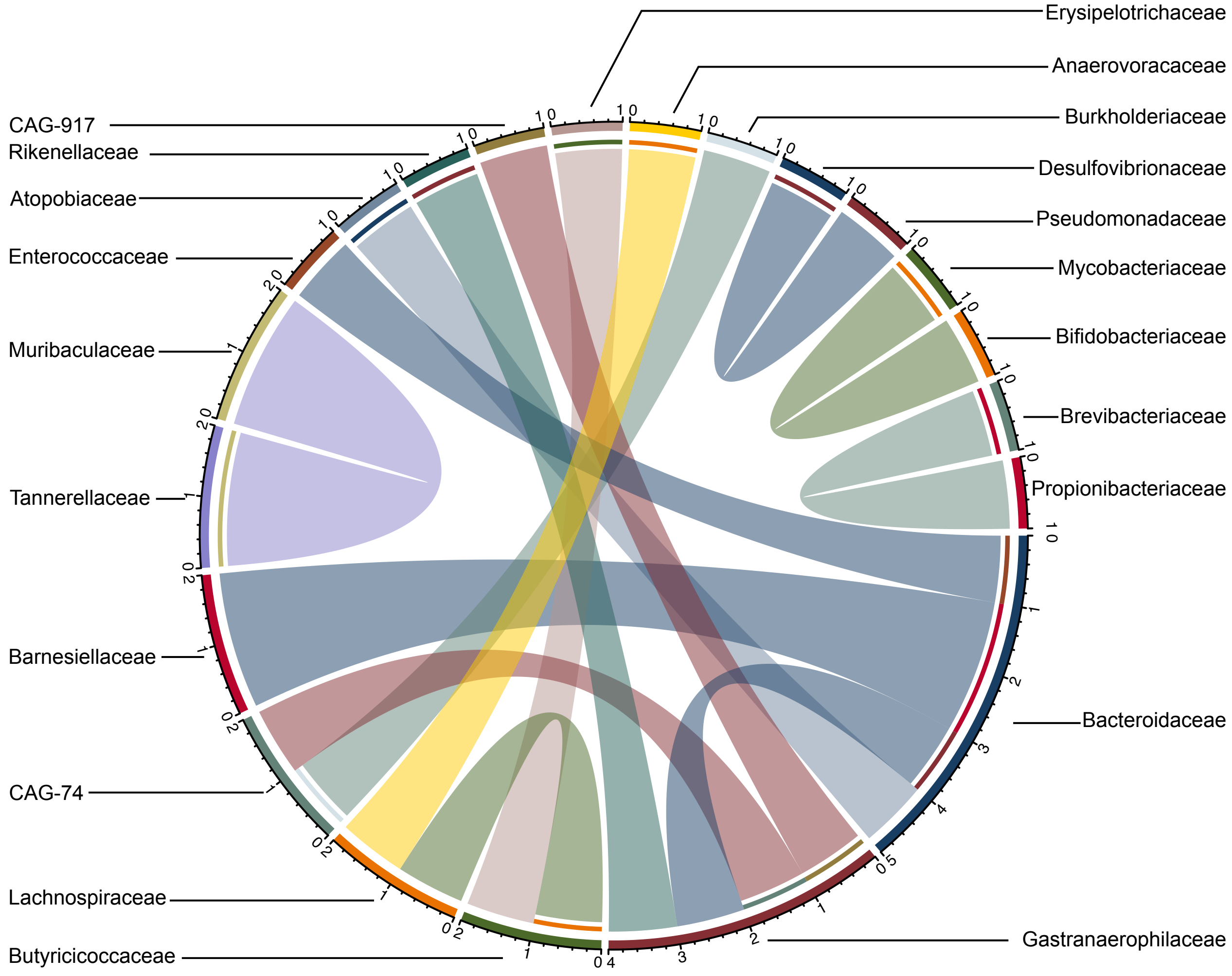


**Supplementary Figure 9: Predicted ARG flow within the chicken gut microbiome at the family level. See Supplementary Figure 8 legend.**

# Supplementary Figure 10

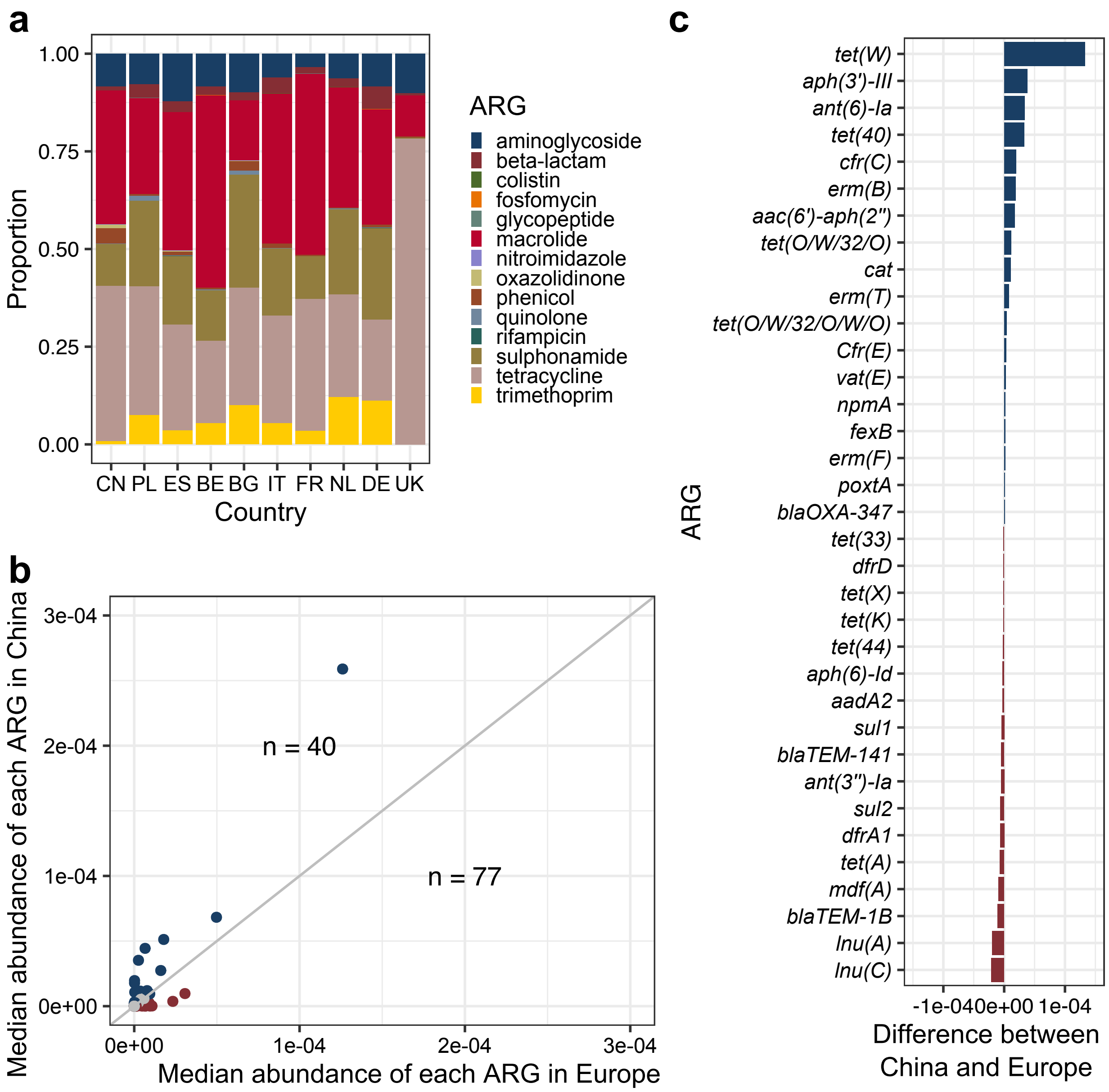


**Supplementary Figure 10: Proportions of ARGs belonging to different categories and difference analyses. (a) Proportions of ARG categories in samples from different countries. (b) Median relative abundances of ARGs in samples from China and Europe. Blue: higher in samples from China, Red: higher in samples from Europe. (c) Major ARGs with differential abundances between China and Europe. Blue bar: higher in China; red bar: higher in Europe (median value over 0.000001, adjusted P < 0.05).**

# Supplementary Figure 11

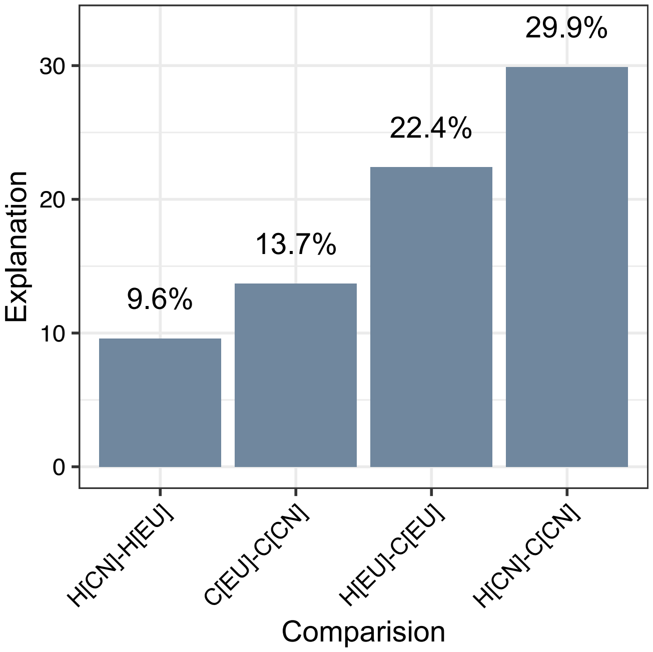


**Supplementary Figure 11: Explanation of host and geographical factors. (*P* < 0.001, adonis, permutations = 999).**
