# Supplementary material for: Metagenome-assembled genomes and gene catalog from the chicken gut microbiome aid in deciphering antibiotic resistomes
Source: Commun Biol. 2021 Nov 18;4:1305. doi: 10.1038/s42003-021-02827-2 (PMC8602611; doi:10.1038/s42003-021-02827-2)
Supplement: Supplementary file 3 — Description of Additional Supplementary Files [file 42003_2021_2827_MOESM3_ESM.pdf]

Description of Additional Supplementary Files

File name: Supplementary Data 1

Description: Metagenomic data available in the present study.

File name: Supplementary Data 2

Description: Statistics of the assembled contigs.

File name: Supplementary Data 3

Description: Basic statistics of the 12,339 MAGs.

File name: Supplementary Data 4

Description: Taxonomy of the 1,978 MAGs at the species level.

File name: Supplementary Data 5

Description: Novel genera assembled in the present study.

File name: Supplementary Data 6

Description: The phylogenetic tree of archaeal genomes at the strain level in newick format.

File name: Supplementary Data 7

Description: Read mapping rates of 799 samples against the 12,339 MAGs.

File name: Supplementary Data 8

Description: Statistics of clean reads in 799 samples.

File name: Supplementary Data 9

Description: The number of MAGs distributed among different countries.

File name: Supplementary Data 10

Description: The number of 535 shared MAGs in different genera.

File name: Supplementary Data 11

Description: The number of genes annotated by the eggNOG and the KEGG in the two gene catalogues.

File name: Supplementary Data 12

Description: The ratio of genes assigned to different COG categories in the two gene catalogues.

File name: Supplementary Data 13

Description: Distribution of plasmids among 477 samples.

File name: Supplementary Data 14

Description: Annotations of major VFs found in the gut microbiome of chickens.

File name: Supplementary Data 15

Description: The number of ARGs found in the 12,339 MAGs.

File name: Supplementary Data 16

Description: Predicted gene flow of ARGs at the family level.

File name: Supplementary Data 17

Description: Coverage of different colistin resistance genes in 799 samples.
