# Supplementary material for: Metagenome-assembled genomes and gene catalog from the chicken gut microbiome aid in deciphering antibiotic resistomes
Source: Commun Biol. 2021 Nov 18;4:1305. doi: 10.1038/s42003-021-02827-2 (PMC8602611; doi:10.1038/s42003-021-02827-2)
Supplement: Supplementary file 21 — Reporting Summary [file 42003_2021_2827_MOESM21_ESM.pdf]

nature portfolio

Corresponding author(s): Yongfei Hu

Last updated by author(s): Oct 14, 2021

Reporting Summary

Nature Portfolio wishes to improve the reproducibility of the work that we publish. This form provides structure for consistency and transparency in reporting. For further information on Nature Portfolio policies, see our Editorial Policies and the Editorial Policy Checklist.

Statistics

For all statistical analyses, confirm that the following items are present in the figure legend, table legend, main text, or Methods section.

n/a	Confirmed	
☐	☒	The exact sample size (*n*) for each experimental group/condition, given as a discrete number and unit of measurement
☐	☒	A statement on whether measurements were taken from distinct samples or whether the same sample was measured repeatedly
☐	☒	The statistical test(s) used AND whether they are one- or two-sided<br>*Only common tests should be described solely by name; describe more complex techniques in the Methods section.*
☐	☒	A description of all covariates tested
☐	☒	A description of any assumptions or corrections, such as tests of normality and adjustment for multiple comparisons
☐	☒	A full description of the statistical parameters including central tendency (e.g. means) or other basic estimates (e.g. regression coefficient) AND variation (e.g. standard deviation) or associated estimates of uncertainty (e.g. confidence intervals)
☐	☒	For null hypothesis testing, the test statistic (e.g. *F*, *t*, *r*) with confidence intervals, effect sizes, degrees of freedom and *P* value noted<br>*Give P values as exact values whenever suitable.*
☐	☒	For Bayesian analysis, information on the choice of priors and Markov chain Monte Carlo settings
☐	☒	For hierarchical and complex designs, identification of the appropriate level for tests and full reporting of outcomes
☐	☒	Estimates of effect sizes (e.g. Cohen's *d*, Pearson's *r*), indicating how they were calculated

Our web collection on statistics for biologists contains articles on many of the points above.

Software and code

Policy information about availability of computer code

Data collection	All the data were downloaded from NCBI or EBI by using aspera (PRJEB33338, PRJEB22062, PRJNA417359 and PRJNA408020).
Data analysis	Software used in the present study: fastp (v0.19.4), Cutadapt (v1.18), BMTagger (v1.1.0), MEGAHIT (v1.1.3), MetaBAT2 (v2.12.1), Maxbin2 (v2.2.6), Concoct (v1.0.0), MetaWRAP (V1.2.1), CheckM (v1.0.12), SPAdes (v3.13.0), dRep (v2.6.2), GTDB-tk (v1.3.0), CompareM (v0.1.2), run_dbCAN2 (v2.0.11), Kraken 2 (v2.0.9-beta), Bracken (v2.6), Prodigal (v2.6.3), BWA-MEM2 (v2.1), SAMtools (v1.11), KofamKOALA (v1.3.0), eggNOG-mapper (v2.0.1b), MetaCHIP (v1.10.0), PhyloPhlAn (v3.0.60), and R (v3.7.3).

For manuscripts utilizing custom algorithms or software that are central to the research but not yet described in published literature, software must be made available to editors and reviewers. We strongly encourage code deposition in a community repository (e.g. GitHub). See the Nature Portfolio guidelines for submitting code & software for further information.

Data

Policy information about availability of data

All manuscripts must include a data availability statement. This statement should provide the following information, where applicable:

- Accession codes, unique identifiers, or web links for publicly available datasets
- A description of any restrictions on data availability
- For clinical datasets or third party data, please ensure that the statement adheres to our policy

The gene catalog and MAGs generated in the present study are available in the National Microbiology Data Center (NMDC, https://nmdc.cn/icrggc/) and the Figshare database with the identifiers “http://10.6084/m9.figshare.15982089” and “http://10.6084/m9.figshare.15911964”).

Field-specific reporting

Please select the one below that is the best fit for your research. If you are not sure, read the appropriate sections before making your selection.

☒ Life sciences ☐ Behavioural & social sciences ☐ Ecological, evolutionary & environmental sciences

For a reference copy of the document with all sections, see nature.com/documents/nr-reporting-summary-flat.pdf

Life sciences study design

All studies must disclose on these points even when the disclosure is negative.

Sample size	No sample-size calculation was performed, We assembled microbial genomes and constructed a gene catalog from 799 chicken gut microbiome samples from countries in China and Europe, which is the number of metagenomic sequencing data from chicken gut microbiome samples we could collected before May 2020.
Data exclusions	The data were excleuded in the present study: 1) lack of geographical information; 2) data were not from the chicken gut microbiome samples. In the downstream analyses, we deleted the sample with less than 15 million paired reads.
Replication	The analyses in the present study were all based on groups with certain number of samples.
Randomization	The allocation was based on the geographical information of each sample.
Blinding	We collected all the chicken gut microbiome samples until May 2020 as far as we know.

Reporting for specific materials, systems and methods

We require information from authors about some types of materials, experimental systems and methods used in many studies. Here, indicate whether each material, system or method listed is relevant to your study. If you are not sure if a list item applies to your research, read the appropriate section before selecting a response.

Materials & experimental systems

n/a	Involved in the study	
☒	☐	Antibodies
☒	☐	Eukaryotic cell lines
☒	☐	Palaeontology and archaeology
☒	☐	Animals and other organisms
☒	☐	Human research participants
☒	☐	Clinical data
☒	☐	Dual use research of concern

Methods

n/a	Involved in the study	
☒	☐	ChIP-seq
☒	☐	Flow cytometry
☒	☐	MRI-based neuroimaging
